# Influence of training status on high-intensity intermittent performance in response to β-alanine supplementation

**DOI:** 10.1007/s00726-014-1678-2

**Published:** 2014-02-06

**Authors:** Vitor de Salles Painelli, Bryan Saunders, Craig Sale, Roger Charles Harris, Marina Yázigi Solis, Hamilton Roschel, Bruno Gualano, Guilherme Giannini Artioli, Antonio Herbert Lancha Jr.

**Affiliations:** 1 Laboratory of Applied Nutrition and Metabolism, School of Physical Education and Sport, University of Sao Paulo, Av. Mello de Moraes, 65-Butanta, São Paulo, SP 05508-030 Brazil; 2 Biomedical, Life and Health Sciences Research Centre, Nottingham Trent University, Nottingham, NG11 8NS UK; 3 Junipa Ltd, Newmarket, Suffolk, UK

**Keywords:** Carnosine, Buffers, Athletes, Dietary supplement, β-Alanine, Performance

## Abstract

Recent investigations have suggested that highly trained athletes may be less responsive to the ergogenic effects of β-alanine (BA) supplementation than recreationally active individuals due to their elevated muscle buffering capacity. We investigated whether training status influences the effect of BA on repeated Wingate performance. Forty young males were divided into two groups according to their training status (trained: T, and non-trained: NT cyclists) and were randomly allocated to BA and a dextrose-based placebo (PL) groups, providing four experimental conditions: NTPL, NTBA, TPL, TBA. BA (6.4 g day^−1^) or PL was ingested for 4 weeks, with participants completing four 30-s lower-body Wingate bouts, separated by 3 min, before and after supplementation. Total work done was significantly increased following supplementation in both NTBA (*p* = 0.03) and TBA (*p* = 0.002), and it was significantly reduced in NTPL (*p* = 0.03) with no difference for TPL (*p* = 0.73). BA supplementation increased mean power output (MPO) in bout 4 for the NTBA group (*p* = 0.0004) and in bouts 1, 2 and 4 for the TBA group (*p* ≤ 0.05). No differences were observed in MPO for NTPL and TPL. BA supplementation was effective at improving repeated high-intensity cycling performance in both trained and non-trained individuals, highlighting the efficacy of BA as an ergogenic aid for high-intensity exercise regardless of the training status of the individual.

## Introduction

Carnosine (β-alanyl-l-histidine) is a cytoplasmic dipeptide found in high concentrations in the skeletal muscle of vertebrates and non-vertebrates, as well as in the central nervous system. Carnosine may play multiple roles in muscle, such as antioxidant (Boldyrev et al. 1993) and anti-glycant (Hipkiss et al. 1995). In addition, recent studies have shown that increased carnosine content in muscle fibres may improve the Ca^2+^ sensitivity of the contractile apparatus and enhance Ca^2+^ release from sarcoplasmic reticulum when Ca^2+^ release is inhibited, such as during fatigue (Dutka et al. 2012). However, it is undisputed that intramuscular pH buffering is a major function of carnosine, a consequence of the pKa of the imidazole ring of carnosine being 6.83 (Bate-Smith 1938).

Harris et al. (2006) showed that β-alanine (BA) availability is the rate-limiting factor for carnosine synthesis in skeletal muscle and that β-alanine supplementation is able to increase the intramuscular content of carnosine. After this seminal study by Harris and colleagues, several investigations have examined the ergogenic effects of β-alanine supplementation on exercise performance and capacity [for a review, see Sale et al. (2013)] and a recent meta-analysis concluded that supplementation significantly improves exercise capacity, but not sport-related performance (Hobson et al. 2012). The body of evidence surrounding the ergogenic effects of β-alanine supplementation is increasing; however, the majority of studies that have shown a positive effect have used recreationally active participants. Although some studies have shown positive effects of β-alanine on trained athletes (Tobias et al. 2013; Salles Painelli et al. 2013; Van Thienen et al. 2009; Derave et al. 2007), several investigations have shown no improvements or marginal effects (Derave et al. 2007; Baguet et al. 2010; Saunders et al. 2012; Hobson et al. 2013; Ducker et al. 2013; Chung et al. 2012; Howe et al. 2014). As such, the ergogenic effects of β-alanine in athletes are less clear than in non-trained individuals. In addition, some authors speculate that athletes might be less responsive to β-alanine supplementation (Bellinger et al. 2012; Bellinger 2014).

It has been shown that trained athletes have an elevated muscle buffering capacity as an adaptation to exercise training (Weston et al. 1997; Edge et al. 2006a, b). Since most studies that recruited athletes have failed to demonstrate an ergogenic effect of β-alanine, it has been speculated that this may be due to the already increased buffering capacity in highly trained individuals, limiting the measurable effect of increased carnosine content through β-alanine supplementation (Bellinger et al. 2012; Bellinger 2014). Although athletic populations are likely to be the most interested in the ergogenic effects of β-alanine, it remains unclear to what extent these highly trained individuals can benefit from supplementation. No study to date has directly compared whether athletes respond differently to β-alanine supplementation in comparison to non-trained recreationally active individuals.

Therefore, to gather knowledge on the potential differential responses to β-alanine supplementation between trained and non-trained participants, the aim of this study was to investigate whether training status influences the ergogenic effect of β-alanine supplementation on repeated Wingate performance using both trained and non-trained cyclists.

## Methods

### Participants

Forty young male individuals participated in this study. They were allocated to two groups according to their training status (endurance-trained cyclists: *N* = 20; non-trained individuals: *N* = 20). Participants were further randomly allocated to the β-alanine and placebo (PL) groups, matched for total work done (TWD) measured during the habituation session. One trained cyclist withdrew from the study due to injury, meaning that 19 trained cyclists were included in the final data set. The cyclists were all actively involved in structured training programs and, although endurance training formed a large part of their training routine, all athletes also undertook regular sprint training. Thirteen of these athletes were competing at national level and the remaining six athletes were participating in state-level official competitions at the time of data collection. The non-trained participants were recreationally active individuals who engaged in a variety of activities (e.g., weightlifting, running, team sports) 1–3 times per week. Participant characteristics, experience and weekly training volumes at the moment they initiated the study are presented in Table 1. The ability to perform high-intensity intermittent exercise was clearly different between the trained and the non-trained groups (Table 2).

**Table 1:** Participant characteristics

	NTPL (*N* = 10)	NTBA (*N* = 10)	TPL (*N* = 9)	TBA (*N* = 10)
Age (years)	26 ± 4	25 ± 4	33 ± 12	32 ± 8
Body mass (kg)	72.6 ± 8.8	77.6 ± 9.9	68.9 ± 10.0	71.7 ± 5.5
Height (m)	1.75 ± 0.08	1.80 ± 0.07	1.79 ± 0.07	1.82 ± 0.05
Weekly training volume (km)	–	–	230 ± 165	278 ± 94
Training experience (years)	–	–	9 ± 6	8 ± 8

*NTPL* non-trained + PL, *NTBA* non-trained + BA, *TPL* trained + PL, *TBA* trained + BA. No differences were observed between groups

**Table 2:** Performance in the four bouts of the Wingate test according to training status

	Non-trained (*N* = 20)	Trained (*N* = 19)	*P*
TWD (J)	49,093 ± 6,043	54,399 ± 6,603	0.013
Performance decrement (%)^a^	32.66 ± 7.38	11.93 ± 6.39	<0.0001
Relative peak power output			
1st bout (W/kg)	8.18 ± 0.70	8.16 ± 0.77	0.940
2nd bout (W/kg)	7.73 ± 0.59	8.03 ± 0.80	0.196
3rd bout (W/kg)	6.89 ± 0.71	7.79 ± 0.69	<0.0001
4th bout (W/kg)	5.99 ± 0.87	7.50 ± 0.66	<0.0001
Relative mean power output			
1st bout (W/kg)	6.67 ± 0.46	6.86 ± 0.51	0.238
2nd bout (W/kg)	5.74 ± 0.42	6.54 ± 0.52	<0.0001
3rd bout (W/kg)	4.93 ± 0.52	6.29 ± 0.50	<0.0001
4th bout (W/kg)	4.48 ± 0.53	6.04 ± 0.51	<0.0001

^a^Decrement in work done from the 1st bout to the 4th bout

Participants were required not to have taken any creatine supplement for 3 months prior to the study and had not taken β-alanine for at least 6 months prior to the study. Participants were also requested to maintain similar levels of physical activity and dietary intake for the duration of the study and compliance with this request was verbally confirmed with participants prior to commencement of the study. Participants were fully informed of any risks and discomforts associated with the study before completing a health screen and providing written consent. The study was approved by the Ethical Advisory Committee from the School of Physical Education and Sport, University of Sao Paulo (Approval Number: 2011/15).

### Experimental design

Participants attended the laboratory on three separate occasions. The first visit was for protocol habituation, with the remaining two visits for the completion of the main trials. One main trial was completed before and one main trial following a 4 week double-blind supplementation period of either β-alanine or placebo. As such, the study comprised four experimental conditions: non-trained + placebo (NTPL, *N* = 10), non-trained + β-alanine (NTBA, *N* = 10), trained + placebo (TPL, *N* = 9) and trained + β-alanine (TBA, *N* = 10).

Participants were supplemented for 4 weeks with either 6.4 g day^−1^ BA (CarnoSyn™, Compound Solutions Inc.™, CA, USA) or an equivalent amount of placebo capsules (dextrose; Ethika Inc.™, São Paulo, Brazil). This dosing regimen has been proven to be effective in increasing muscle carnosine content over 2 (Stellingwerff et al. 2012) and 4 weeks (Harris et al. 2006), and it is ~10–20 times higher than the average β-alanine intake from food, as calculated for the participants of the present study (results are shown in Table 3). The participants took two 800-mg gelatin capsules four times per day at 3–4 h intervals. Carboxymethyl cellulose was added to the β-alanine capsules (100 mg per 800 mg of β-alanine) in order to slow the absorption of β-alanine and minimize paraesthesia. Participants received exactly 4 weeks’ worth of supplement in a sealed, unmarked container, and compliance was monitored by verifying the remaining contents of the container upon return to the lab following the supplementation period. The degree of compliance was reported to be 100 % in all groups.

**Table 3:** Energy, macronutrient and β-alanine intake

	NTPL (*N* = 10)	NTBA (*N* = 10)	TPL (*N* = 9)	TBA (*N* = 10)
Energy (Kcal)	2,747 ± 661	3,364 ± 662	3,181 ± 430	2,511 ± 769
Carbohydrate (g)	342 ± 96	448 ± 86	364 ± 50	304 ± 64
Protein (g)	134 ± 37	150 ± 39	140 ± 42	111 ± 39
Fat (g)	94 ± 35	108 ± 47	129 ± 23	95 ± 52
β-Alanine (mg)	383 ± 191	397 ± 192	446 ± 328	364 ± 283

### Experimental procedures

#### Preliminary testing

Height and body mass were recorded upon arrival at the laboratory. The exercise protocol required participants to complete 4 bouts of a modified lower-body cycling Wingate test (Inbar and Bar-Or 1986), using a specifically designed ergometer (Biotec 2100, Cefise, Brazil). The position on the cycle ergometer was determined prior to the habituation session, recorded and maintained for all subsequent trials. The participants’ feet were securely attached to the pedals using toe clips and straps. Participants warmed up for 5 min on the ergometer prior to the first bout, against no resistance. On completion of the warm up, the test began immediately from a static start. Each Wingate bout lasted 30 s and participants were required to cycle against a load set at 5 % of body mass, which was measured with a digital scale to the nearest 10 g. This 5 % load was chosen over the original 7.5 % load in order to allow the non-trained participants to complete the entire 4-bout protocol. Bouts were interspersed by 3-min passive recovery, with all the participants being seated during this recovery period. Standardised verbal encouragement was given throughout every bout. Upon completion of the test, the resistance was removed and the participants were instructed to continue cycling at a self-selected cadence to facilitate recovery. A set of 24 sensors measured wheel velocity, with power output being calculated automatically every second by computer software (Ergometric 6.0, Cefise, Brazil). Mean power (MPO, W) and peak power (PPO, W) output were obtained for each bout and TWD (J) was obtained for the overall test session. Performance decrement was measured as the percentage loss in work done from the 1st to the 4th bout. The coefficient of variation for TWD in the non-trained and the trained groups was 2.42 ± 1.86 and 1.78 ± 1.27 %, respectively.

#### Main trials

Participants were requested to abstain from alcohol and strenuous exercise in the 24-h period prior to the main trials. Participants arrived at the laboratory in at least 2 h following their last meal, and immediately began their warm up followed by the 4-bout lower-body cycling Wingate test. *Ad libitum* water consumption was allowed during the main trials. To control for intervening variables, food intake was assessed during the supplementation period by means of three 24-h dietary recalls undertaken on separate days (2 weekdays and 1 weekend day), with the aid of a visual photo album of real-sized foods and portions. Nutritional supplements were also recorded. Energy and macronutrient intakes were analysed by the software Virtual Nutri™ (São Paulo, Brazil). The intake of β-alanine from fish and red meats containing carnosine and its related derivatives was estimated from the data of Jones et al. (2011).

### Statistical analyses

All data are presented as mean ± SD and were analysed using the SAS statistical package, (SAS version 9.2). A one-way ANOVA, with Group (i.e. NTPL, NTBA, TPL and TBA) as a fixed factor, was used to compare the participant characteristics and baseline performance between groups. The mixed models for repeated measures were used to examine the effect of supplementation on TWD, with Group and Time (i.e. pre-supplementation, post-supplementation) as fixed factors. To mitigate the impact of inter-individual data variability, all TWD values were converted into delta scores (i.e., POST-PRE values) and thereafter tested by a mixed model assuming “pre values” as a covariate. The mixed models were also performed to examine the effect of supplementation on MPO and PPO, with Group, Time and Bout (i.e. bout 1, bout 2, bout 3, bout 4) as fixed factors. Tukey tests were used for post hoc analyses and effect sizes were calculated using Cohen’s d. Statistical significance was accepted at *p* ≤ 0.05, with a trend towards significance being accepted at *p* ≤ 0.1.

## Results

### Wingate test

#### Effects of β-alanine supplementation regardless of training status

**Fig. 1:**
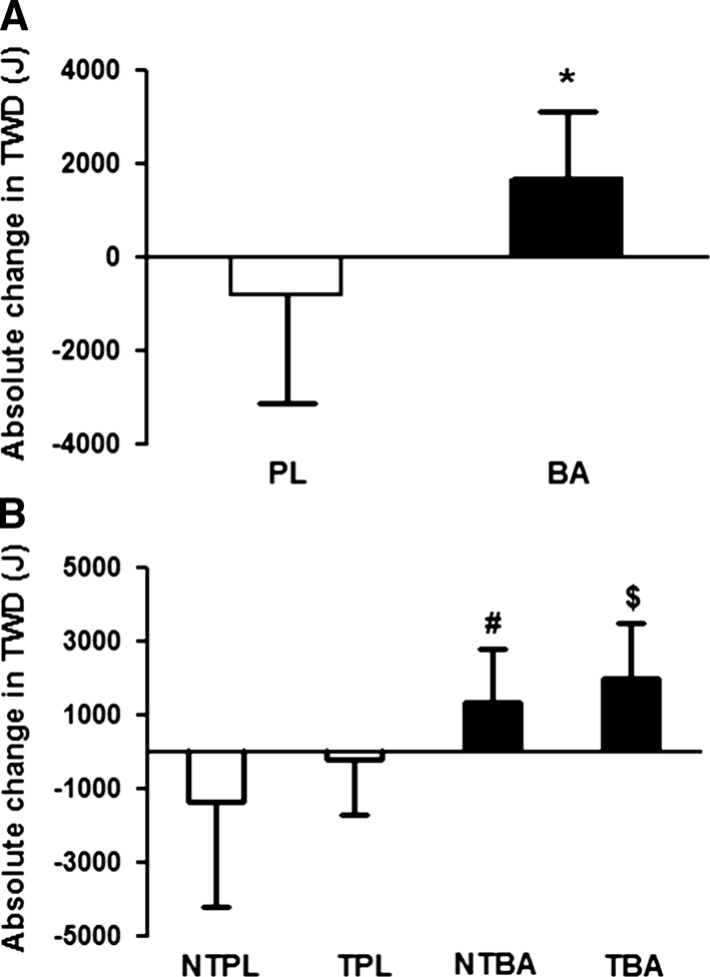
**a** Absolute change in performance in placebo (*PL*) and β-alanine (*BA*) with trained and non-trained participants grouped together. *p ≤ 0.001 from placebo. **b** Absolute change in performance for individual groups NTPL, TPL, NTBA and TBA. ^#^ *p* = 0.008 from NTPL. ^$^ *p* = 0.037 from TPL. Effect sizes: NTPL—0.2; TPL—0.03; NTBA—0.2; TBA—0.4

**Fig. 2:**
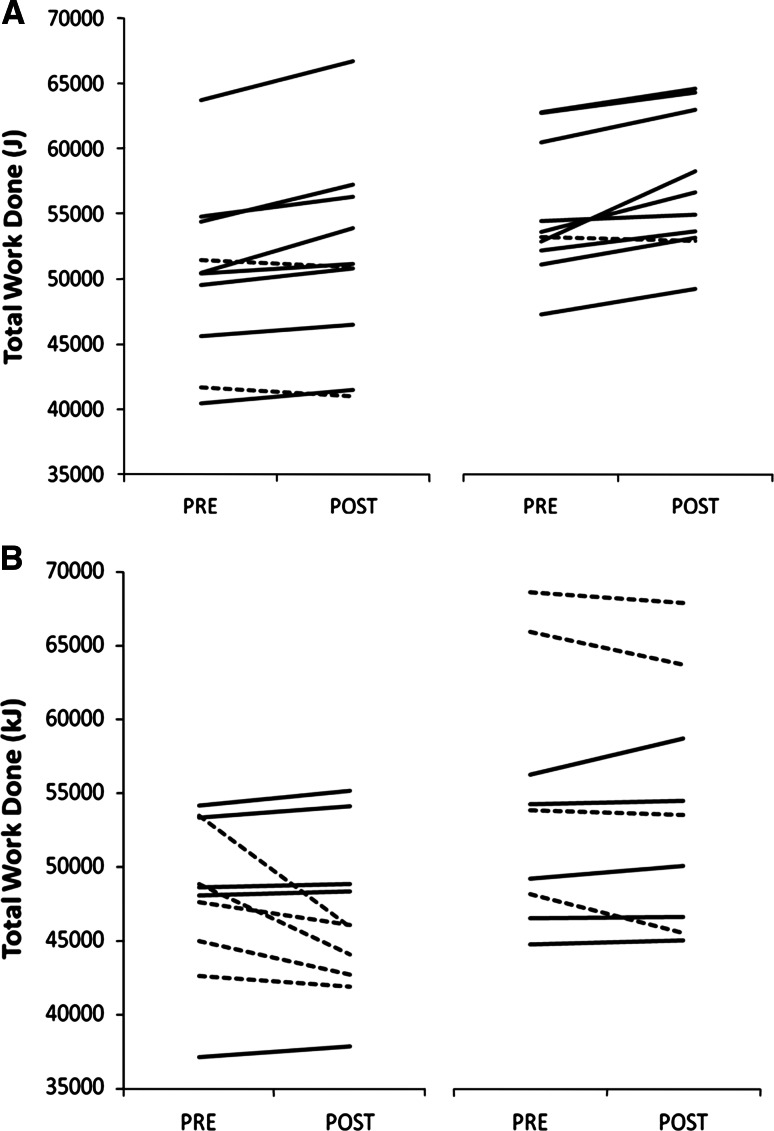
**a** Individual response in TWD to supplementation in the β-alanine groups (NTBA and TBA, respectively). **b** Individual response in TWD to supplementation in the placebo groups (NTPL and TPL, respectively). Individuals who improved performance are indicated by a *solid line*, while those who did not are indicated by a *dotted line*

There was no difference in TWD between placebo and β-alanine groups prior to supplementation (placebo: 50,773 ± 7,349 J; β-alanine: 52,538 ± 6,283 J; *p* = 0.44). There was a significant interaction effect of Group × Time on TWD (*F* = 16.48; *p* = 0.0002); following supplementation, TWD was increased by 1,663 ± 1,457 J in the β-alanine group (*p* = 0.0004) and decreased by 832 ± 2,308 J in the placebo group (*p* = 0.07; Fig. 1a). A total of 17 out of the 20 participants supplemented with β-alanine improved TWD following supplementation; 8 out of the 10 participants in the NTBA improved their performance, while 9 out of the 10 in the TBA group showed improved performance (Fig. 2a). On the other hand, only 10 out of the 19 participants supplemented with placebo improved TWD following supplementation; 5 out of the 10 participants in the NTPL improved their performance, while 5 out of the 9 in the TPL group showed improved performance (Fig. 2b).

#### Effects of β-alanine supplementation in trained versus non-trained subjects

Prior to supplementation, performance decrement from the 1st to the 4th Wingate bout was 32.3 ± 6.6 and 33.1 ± 8.5 % for the NTPL and the NTBA groups, respectively, with no differences between these two groups (*p* > 0.05). For the trained athletes, prior to the supplementation period, the performance decrement in the Wingate was 11.6 ± 7.9 and 12.3 ± 5.0 % for the TPL and the TBA, respectively (*p* > 0.05). Performance decrement for the NTPL and the NTBA groups was significantly higher when compared with both the TPL and the TBA groups (all *p* < 0.05).

TWD did not differ within the non-trained (NTPL: 47,913 ± 5,313 J and NTBA: 50,274 ± 6,765 J; *p* = 0.43) and trained groups (TPL: 53,951 ± 8,258 J and TBA: 54,802 ± 5,119 J; *p* = 0.78) prior to supplementation. TWD was significantly increased following supplementation in both the NTBA (+1,349 ± 1,411 J; *p* = 0.03) and TBA groups (+1,978 ± 1,508 J; *p* = 0.002), and it was significantly reduced in the NTPL (−1,385 ± 2,815 J; *p* = 0.03) with no significant difference being shown in the TPL group (−219 ± 1,507 J; *p* = 0.73). Analysis of covariance revealed that absolute change in TWD in both supplemented groups was significantly higher than in their respective placebo groups (NTBA vs. NTPL: *p* = 0.008; TBA vs. TPL: *p* = 0.037). However, no significant differences were shown between groups (NTPL vs. TPL: *p* = 0.10; NTBA vs. TBA: *p* = 0.60) indicating that the effect of β-alanine supplementation was not affected by training status (Fig. 1b).

**Fig. 3:**
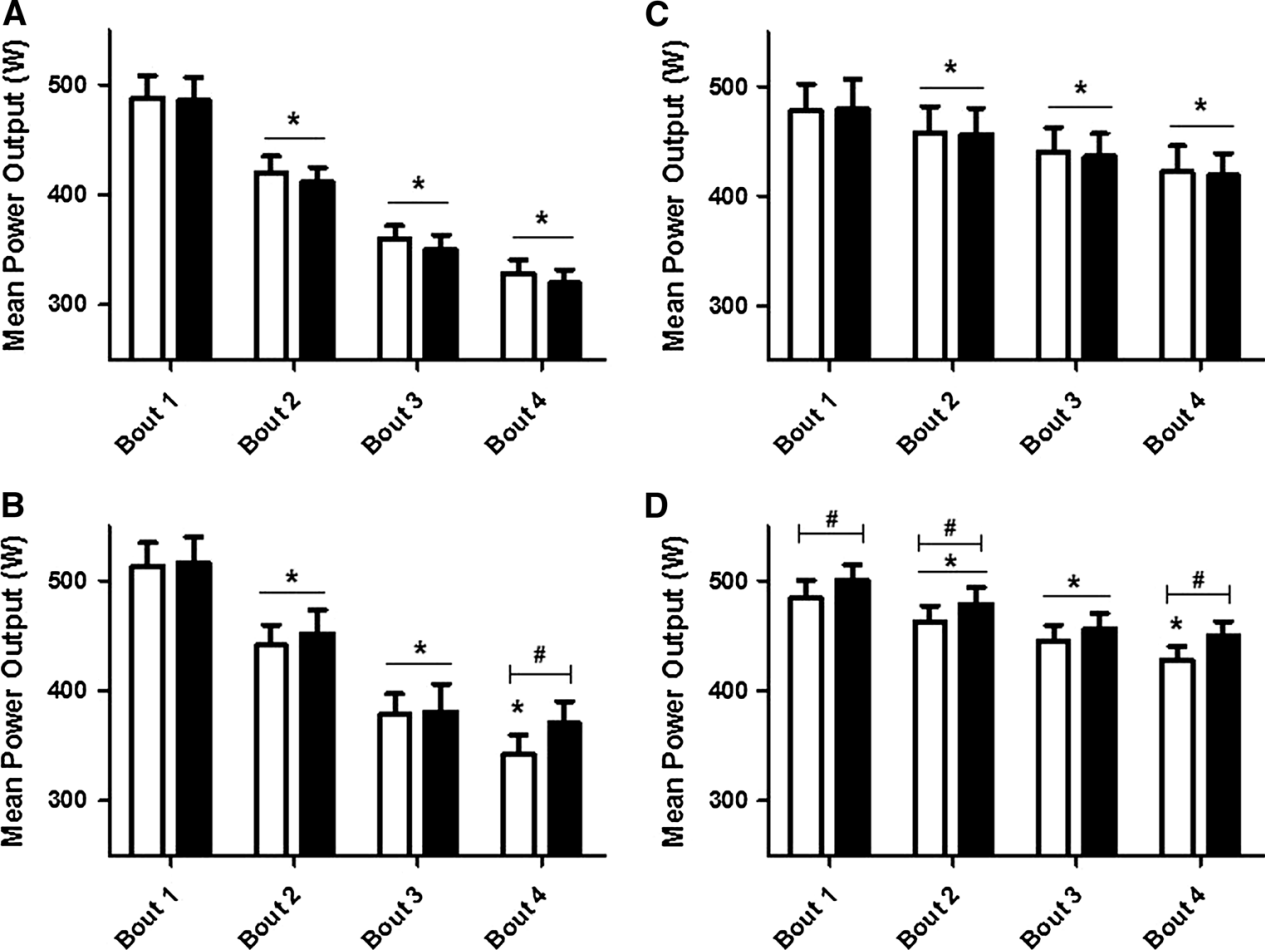
MPO during each bout of the Wingate test for NTPL (**a**), NTBA (**b**), TPL (**c**), and TBA (**d**). Pre- and post-supplementation MPO are indicated by *white* and *black bars*, respectively. *p ≤ 0.05 from the previous bout. ^#^ *p* ≤ 0.05 from pre-supplementation during the same bout

MPO was reduced in each subsequent bout during the pre-supplementation trial for all of the groups (Fig. 3). There was an interaction effect on MPO (Group × Time × Bout, *F* = 4.89; *p* < 0.0001); post hoc tests revealed that both supplemented groups (i.e. NTBA and TBA) were able to maintain MPO from bouts 3–4 following supplementation (both *p* > 0.05). Compared to the pre-supplementation trial, post-supplementation MPO was higher in bout 4 for the NTBA (*p* = 0.0004), and higher in bouts 1, 2 and 4 for the TBA group (*p* ≤ 0.05, Fig. 3).

In the pre-supplementation test, both of the non-trained groups (i.e. NTPL and NTBA) showed a significant reduction in PPO in every subsequent trial (all *p* < 0.05). Conversely, both trained groups were able to maintain PPO across bouts pre-supplementation (all *p* > 0.05). Importantly, there was an interaction effect on PPO (Group × Time × Bout, *F* = 2.70; *p* = 0.003), with increased values shown post-supplementation in bout 4 for the NTBA (*p* = 0.004); and a tendency for increased values in bout 2 for the TBA group (*p* = 0.08).

### Food intake

The energy, carbohydrate, protein, fat and β-alanine intake are presented in Table 3. No significant differences were observed within or between groups (all *p* > 0.05).

### Blinding efficacy and side effects

Of the 20 participants who were supplemented with β-alanine, only 4 were able to correctly guess their supplement. Of the 19 participants who were supplemented with dextrose, only 9 were able to correctly guess their supplement (Fischer exact test: *p* = 0.33). Three participants taking β-alanine reported mild paraesthesia and were able to correctly identify their supplement. We reanalysed the data without these three participants and the results remained similar, with no significant differences in the overall findings (as such, these data are not shown). No side effects related to dextrose ingestion were reported.

## Discussion

Since there is the possibility that an already increased muscle buffering capacity explains the lack of a measurable ergogenic effect of β-alanine in trained athletes, we chose, for the first time, to directly investigate whether training status influences the ergogenic effect of β-alanine supplementation. The main findings of this study were that β-alanine supplementation improved repeated-bout high-intensity cycling performance, to a similar extent, in both trained and non-trained participants.

The findings of our study are in contrast to previous studies that have shown no effect of β-alanine on highly trained individuals (Derave et al. 2007; Baguet et al. 2010; Bellinger et al. 2012; Saunders et al. 2012). The differences in results might be due to the protocols employed; previous studies may not have used exercise tests of sufficient intensity or duration to be influenced by decreased intramuscular pH, or may have been susceptible to pacing strategies, masking any ergogenic effects of increased muscle buffering capacity. Intermittent supra-maximal exercise promotes a considerably greater intramuscular acidosis than continuous high-intensity exercise (Hermansen and Osnes 1972; Belfry et al. 2012), probably because the former is more reliant on glycolytic ATP resynthesis (Belfry et al. 2012). Thus, it is reasonable to assume that intermittent exercise performance is more likely to be limited by muscle acidosis and, hence, more susceptible to improvements with increased buffering capacity. The 4-bout lower-body modified Wingate test is extremely high-intensity in nature and requires the individual to perform maximally in every bout. Artioli et al. (2007) and De Salles Painelli et al. (2014) showed blood lactate values up to ~15 mmol L^−1^ following the 4-bout upper-body Wingate test and venous blood pH as low as ~7.05 following 3 bouts of the upper-body Wingate test, highlighting the relevant contribution of anaerobic glycolysis for tests similar in duration, number of bouts and recovery between bouts. Therefore, the repeated-bout Wingate test would appear to be an appropriate model to detect performance improvements promoted by nutritional strategies that increase buffering capacity.

In the present study, TWD did not differ within the non-trained (NTPL vs. NTBA) and trained groups (TPL vs. TBA) prior to supplementation, indicating that randomisation successfully generated similar groups in each profile. Moreover, TWD prior to supplementation in the trained participants was ~11 % higher (*p* < 0.05) than in the non-trained participants, and work decrement from the 1st to the 4th bout in the non-trained participants was 20 % higher than in their trained counterparts (average 32 vs. 12 % decrement, respectively; *p* < 0.05). Since it has been demonstrated that the buffering capacity of homogenates of muscle (which is assumed to be a measure of muscle buffering capacity in situ) is related to repeated sprint ability and work decrement over repeated sprints (Bishop et al. 2004), it is plausible to assume that individuals in the trained groups have higher muscle buffering capacity than those in the non-trained groups. This, however, was not directly measured in the present investigation because it would require muscle biopsies, which are excessively invasive to be performed in athletes in the middle of the competitive season.

It should be noted that the peak and mean power attained in the 1st bout were not different between trained and non-trained. However, this was most likely caused by the lowered mechanical load of the Wingate test (5 % in the present study instead of the original 7.5 %) employed in our protocol, which hampered the Wingate test to distinguish the trained from the non-trained participants. On the other hand, this modification was necessary to allow the untrained individuals to complete the entire 4-bout protocol, which would not be possible for most of them if we used the 7.5 % load. In spite of the lack of differences in the first bout, our 4-bout modified Wingate test was able to clearly differentiate both groups in terms of mean and peak power in the last 2–3 bouts, TWD in the overall protocol and performance decrement over the 4 bouts.

Despite the remarkable differences in repeated sprint ability between the trained and non-trained individuals, four weeks of β-alanine supplementation increased TWD to a similar extent in both groups (non-trained: 2.52 ± 2.64 %, trained: 3.64 ± 2.87 %). The magnitude of the improvement in performance observed in the present study is in agreement with a recent meta-analysis that showed a 2.85 % positive effect of β-alanine (Hobson et al. 2012). Although improvements in TWD around 3.0 % may suggest only a discrete effect, it is worth noting that TWD in the trained participants was ~11 % higher than in their non-trained counterpart, meaning that 4 weeks of supplementation elicited a performance improvement which represents about one-third of the improvement achieved by several years of training.

In the present study, TWD increased due to an increased ability to maintain MPO during the Wingate bouts. A beneficial effect can be expected in the latter bouts of the test, since a single 30-s maximal effort is unlikely to be affected by reduced intramuscular pH (Bogdanis et al. 1998). Artioli et al. (2007) employed an upper-body Wingate protocol to determine the effect of increased extracellular buffering and showed an increase in MPO in the last bout of exercise when experienced judo athletes were supplemented with sodium bicarbonate. Similarly, the non-trained participants supplemented with β-alanine in this study improved their MPO in the final bout of exercise, likely due to an increase in muscle buffering capacity over the exercise pH transit range, from increased carnosine concentrations. However, the trained participants supplemented with β-alanine showed an improved MPO across almost all of the bouts. This may be due to the training status of the trained athletes, who are likely to have had an increased ability to consistently perform maximal exercise across each bout, resulting in an earlier intramuscular H^+^ accumulation, placing an earlier reliance on their buffering capacity. Furthermore, it is possible that β-alanine supplementation provided a higher training quality during the supplementation period, enhancing training volume and/or intensity, thus increasing MPO in almost all of the bouts in TBA.

A potential limitation of this study is that we were unable to perform muscle analyses to confirm the efficacy of β-alanine in increasing muscle carnosine content. However, all human studies using 1.6–6.4 g day^−1^ of β-alanine for 4 weeks or longer have so far reported increases of at least 8 mmol kg^−1^ dry muscle (corresponding to an increase of 40 % in muscle carnosine) (Sale et al. 2013). According to the estimates by Harris et al. (2006), a 40 % increase in muscle carnosine would represent a ~4 % increase in whole muscle buffering capacity and a ~5 % in type II fibres buffering capacity. Considering that all human studies with β-alanine supplementation have shown increased muscle carnosine, it appears to be safe to assume that our β-alanine supplementation protocol resulted in increased muscle carnosine. Also, based on the current findings showing that trained and non-trained participants equally improved their performance, it is reasonable to assume that carnosine accrual after β-alanine supplementation is not influenced by training status and training history. This is in agreement with Kendrick et al. (2009) using the same supplementation protocol as used in the present study and where the carnosine content of types I and II muscle fibres was measured directly, but in contrast with a recent study by Bex et al. (2014). The latter study, based on MRS measurements, suggested that carnosine elevation induced by β-alanine is higher in trained as compared to non-trained muscles. Further studies directly comparing the responses of both trained and non-trained individuals to β-alanine supplementation and its effects on performance and muscle carnosine content in different muscle fibre types are needed to clarify this issue.

It is important to note that 3 out of 20 of the participants ingesting β-AThe energy, carbohydrate, protein, fat and β-alaninelanine reported mild paraesthesia, which is a symptom commonly described when a single dose of 1,600 mg is ingested (Harris et al. 2006; Décombaz et al. 2012). The same symptoms are not observed when 1,600 mg of β-alanine is provided in controlled release tablets (Saunders et al. 2012; Décombaz et al. 2012), suggesting that the strategy adopted in our study to slow the absorption of β-alanine (i.e., the addition of carboxymethyl cellulose added to the gelatin capsules) was not totally successful in preventing paraesthesia. Despite this, our data show that the participants as a group were not able to correctly guess the substance that they were ingesting, indicating that the blinding of the present study remained intact.

It can be concluded that 4 weeks of β-alanine supplementation improved repeated high-intensity cycling performance in both trained and non-trained participants. The findings of the present study indicate the efficacy of β-alanine as an ergogenic aid for high-intensity intermittent exercise regardless of the training status, highlighting that highly trained athletes can benefit from the use of β-alanine.
